# Analyzing Age-Related Macular Degeneration Progression in Patients with Geographic Atrophy Using Joint Autoencoders for Unsupervised Change Detection

**DOI:** 10.3390/jimaging6070057

**Published:** 2020-06-29

**Authors:** Guillaume Dupont, Ekaterina Kalinicheva, Jérémie Sublime, Florence Rossant, Michel Pâques

**Affiliations:** 1 ISEP, DaSSIP Team, 92130 Issy-Les-Moulineaux, France; guillaume.dupont@isep.fr (G.D.); ekaterina.kalinicheva@isep.fr (E.K.); 2 Université Paris 13, LIPN - CNRS UMR 7030, 93430 Villetaneuse, France; 3 Clinical Imaging Center 1423, Quinze-Vingts Hospital, INSERM-DGOS Clinical Investigation Center, 75012 Paris, France; mpaques@15-20.fr

**Keywords:** ARMD, change detection, unsupervised learning, medical imaging

## Abstract

Age-Related Macular Degeneration (ARMD) is a progressive eye disease that slowly causes patients to go blind. For several years now, it has been an important research field to try to understand how the disease progresses and find effective medical treatments. Researchers have been mostly interested in studying the evolution of the lesions using different techniques ranging from manual annotation to mathematical models of the disease. However, artificial intelligence for ARMD image analysis has become one of the main research focuses to study the progression of the disease, as accurate manual annotation of its evolution has proved difficult using traditional methods even for experienced practicians. In this paper, we propose a deep learning architecture that can detect changes in the eye fundus images and assess the progression of the disease. Our method is based on joint autoencoders and is fully unsupervised. Our algorithm has been applied to pairs of images from different eye fundus images time series of 24 ARMD patients. Our method has been shown to be quite effective when compared with other methods from the literature, including non-neural network based algorithms that still are the current standard to follow the disease progression and change detection methods from other fields.

## 1. Introduction

**Figure 1:**
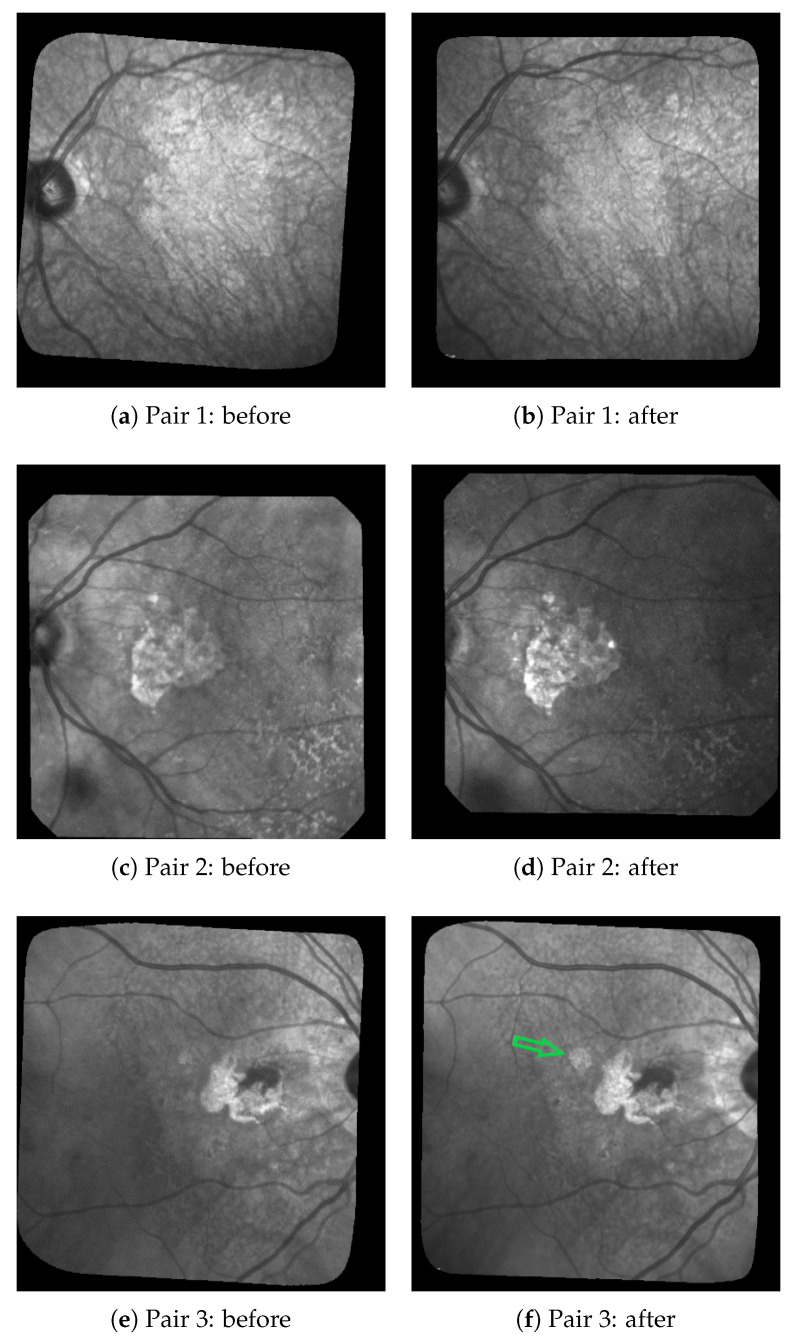
3 of pairs of images acquired six months apart, the GA corresponds to the bright areas. The green arrow in (**f**) shows a new lesion.

Dry age-related macular degeneration (ARMD or sometimes AMD), a degenerative disease affecting the retina, is a leading cause of intractable visual loss. It is characterized by a centrifugal progression of atrophy of the retinal pigment epithelium (RPE), a cellular layer playing a key role in the maintenance of the photoreceptors. Blindness may occur when the central part of the eye, the fovea, is involved. The disease may be diagnosed and monitored using fundus photographs: ophthalmologists can observe pathologic features such as drusen that occur in the early stages of the ARMD, and evaluate the geographic atrophic (GA) progression in the late stages of degeneration (Figure 1).

Automatic analysis of fundus images with dry ARMD is of high medical interest [1] and this has been an important research field for two decades, for diagnosis [2] or follow up [3,4] purposes. Imaging modalities are most often color eye fundus images [5,6,7], fundus autofluorescence (FAF) [4,8,9], and, to a lesser extent, confocal scanning laser ophthalmoscopy (cSLO) in infrared (IR), or optical coherence tomography (OCT) [10]. In this work, we process cSLO images in IR: this modality is comfortable for the patient, and it has higher resolution and higher contrast than color imaging, an older technology. Our goal is to detect the appearance of new atrophic areas and quantify the growth of GA from pairs of images acquired at regular time intervals to ultimately propose predictive models of the disease progress.

Figure 1 shows three pairs of consecutive images, taken at 6-month intervals. The lesions (GA) are the brighter regions in the fundus and around the optical disk. Automatic processing to follow up these areas is obviously very challenging given the quality of the images: uneven illumination, saturation issues, illumination distortion between images, GA poorly contrasted with retinal structures interfering (vessel, optical disk), blur, etc. The difficulty also lies in the high variability of the lesions in terms of shape, size, and number. The lesion boundary is quite smooth in some cases (c and d) and very irregular in others (a and b). At any time, new spots can appear (as shown by the green arrow between e and f) and older lesions can merge. All these features make the segmentation task very difficult, and especially long and tedious to perform manually. It is worth noting that even experts cannot be sure of their manual delineation in all cases.

Modeling ARMD evolution from a series of eye fundus images requires segmenting the GA in each image and/or to perform a differential analysis between consecutive images to get the lesion growth. In this paper, we propose a differential analysis method based on a joint convolutional fully convolutional autoencoder. Our model is fully unsupervised and does not require labeled images that are difficult to come by in quantity and quality high enough to train a supervised neural network. Our method is applied to pairs of images of a patient eye fundus time series and aims at efficiently segmenting medically significant changes between the two images: meaningless differences caused by image quality or lighting issues are ignored while changes related to GA lesion evolution are extracted.

The remainder of the paper is organized as follows: In Section 2, we review works dedicated to automated processing of images of eye fundus with ARMD and we present approaches for change detection applied to medical image analysis as well as other fields. In Section 3, we present briefly our data and the way we process our images. Then, we detail our proposed method and the architecture of our neural network (Section 4). Section 5 shows our experimental results. Finally, we draw some conclusions in Section 6 and give some possible future perspectives of this work.

## 2. Related Works

This state-of-the-art section is split into three sections presenting methods closest to this work and also based on the differential approach first. Then, we will introduce a few methods also developed for the study of ARMD or other eye diseases but that rely on a segmentation based approach on individual images. In addition, we will finish this state of the art with some examples of other change detection methods from outside the field of medical imaging.

Before we start, we remind our readers that this work introduces a fully unsupervised method for change detection in ARMD images. The main difference between supervised and unsupervised learning is the following:

- In supervised learning, Machine Learning algorithms are trained using data that have been pre-classified or annotated by human with the goal of building a model based on these data. This model is then applied to new data with the goal of providing a classification for them. While this type of Machine Learning is considered to be the most powerful, its main weakness is that it cannot be used if no or not enough annotated data are available.
- In unsupervised learning, on the other hand, all data are provided raw and without any annotation or classification, and the algorithm must find by itself different classes called clusters of elements that are similar. Since the process is not guided, hence the name “unsupervised”, the cluster found using this process may or may not match the classes expected by the users, and the performances of unsupervised learning are expected to be lower than these of supervised learning. Unsupervised Learning is usually used as an exploratory task or when there are no available annotated data (or not enough), both of which are the case for our application to ARMD image time series.

With this related work section, we hope to demonstrate that providing fully automated tools reaching the required level of performance for medical application is a difficult task, especially in an unsupervised context with few annotated images.

### 2.1. Differential Approaches Applied to ARMD

The following works are most related to our proposed algorithm as they are unsupervised algorithms applied to various eye disease images, including ARMD: In [11], Troglio et al. published an improvement of their previous works realized with Nappo [12] where they use the Kittler and Illingworth (K&I) thresholding method. Their method consists of applying the K&I algorithm on random sub-images of the difference image obtained between two consecutive eye fundus images of a patient with retinopathy. By doing so, they obtain multiple predictions for each pixel and can then make a vote to decide the final class. This approach has the advantage that it compensates for the non-uniform illumination across the image; however, it is rather primitive since it does not actually use any Machine Learning and rely on different parameters of the thresholding method to then make a vote. To its credit, even if it achieves a relatively weak precision, it is fully unsupervised like our method. In [6], the authors tackle a similar problematic to ours where they correct eye fundus images by pairs, by multiplying the second image by a polynomial surface whose parameters are estimated in the least-squares sense. In this way, illumination distortion is lessened and the image difference enhances the areas of changes. However, the statistical test applied locally at each pixel is not reliable enough to get an accurate map of structural changes.

### 2.2. Segmentation First Approaches Applied to ARMD and Other Eye Diseases

Other works related with eye diseases take the different approach of segmenting lesions in individual images instead of looking for changes in pairs of images. In [5], Köse et al. proposed an approach where they first segment all healthy regions to get the lesions as the remaining areas. This approach also requires segmenting separately the blood vessels, which is known to be a difficult task. This method involves many steps and parameters that need to be supervised by the user. In [4], Ramsey et al. proposed a similar but unsupervised method for the identification of ARMD lesions in individual images: They use an unsupervised algorithm based on fuzzy c-means clustering. Their method achieves good performances for FAF images, but it performs less well for color fundus photographs. We can also mention the work of Hussain et al. [13] in which the authors are proposing another supervised algorithm to track the progression of drusen for ARMD follow-up. They first use U-Nets to segment vessels and detect the optic disc with the goal of reducing the region of interest for drusen detection. After this step, they track the drusen using intensity ratio between neighbor pixels.

Using the same approach of segmenting the lesions first and then tracking the changes, there are a few supervised methods available. We can, for instance, mention [14] in which the authors propose another related work in which they use a pre-trained supervised neural network to detect ARMD lesions with good results. In addition, in [15], the same team uses another supervised convolutional neural network (CNN) to assess the stage of ARMD based on the lesions.

Other traditional more machine learning approaches have also been used for GA segmentation such as the k-nearest neighbor classifiers [9], random forests [7], as well as combinations of Support Vector Machines and Random Forests [16]. Feature vectors for these approaches typically include intensity values, local energy, texture descriptors, values derived from multi-scale analysis and distance to the image center. Nevertheless, these algorithms are supervised: they require training the classifier from annotated data, which brings us back to the difficulty of manually segmenting GA areas.

Related to other medical images, in [17], the authors show that the quantization error (QE) of the output obtained with the application of Self Organized Maps [18] is an indicator of small local changes in medical images. This work is also unsupervised but has the defaults that the SOM algorithm cannot provide a clustering on its own and must be coupled with another algorithm such as K-Means to do so. Furthermore, since there is no feature extraction done, this algorithm would most likely be very sensitive to the lighting and contrast issues that are present in most eye fundus time series. Lastly, the use of SOM based methods on monochromatic images is discouraged since no interesting topology may be found from a single attribute.

Finally, the literature also contains a few user-guided segmentation frameworks [19,20] that are valuable when it is possible to get a user input.

### 2.3. Change Detection Methods from Other Fields

Apart from medicine, change detection algorithms have been proposed for many different applications such as remote sensing or video analysis. In [21], the authors reveal a method combining principal component analysis (PCA) and K-means algorithm on the difference image. In [22], an architecture relying on joint auto-encoders and convolutional neural networks is proposed to detect non-trivial changes between two images. In [23], the authors propose an autoencoder architecture for anomaly detection in videos.

Finally, as we have seen that quite a few methods rely on segmentation first and change detection after, we can also mention a few noteworthy unsupervised segmentation algorithms used outside the field of medicine: Kanezaki et al. [24] used CNN to group similar pixels together with consideration of spatial continuity as a basis of their segmentation method. In addition, Xia and Kulis [25] developed W-Net using a combination of two U-Nets with a soft Normalized-Cut Loss.

In Table 1, we sum up the main methods presented in this related work section. The first column specifies if the method is supervised or unsupervised. The second column indicates if the method uses the directly pairs of images (or their difference), or if it uses individual images to segment them first and then compare the segmentations. The “Algorithm” column details which algorithm is used. The last column gives the application field.

## 3. Dataset Presentation

In this section, we will provide some details on the image time series used in this work in terms of their characteristics, flaws, and how we pre-processed them before comparing different methods for change detection on them.

Our images whose main characteristics can be found in Table 2 were all acquired at the Quinze–Vingts National Ophthalmology Hospital in Paris, in cSLO with IR illumination. This modality has the advantage of being one of the most common and cheapest legacy method of image acquisition for eye fundus images, thus allowing to have lots of images and to follow the patients for several years. However, it is infrared only and therefore all images are monochromatic and may contain less information than images acquired from other techniques with multiple channels (that are less common for this type of exam and more difficult to find in numbers).

While some 3D OCT and 2D FAF images were available, the infrared light penetrates better than blue light through media opacities and requires pupil dilation hence IR imaging is more robust (and better supported by patients). Although OCT is becoming the preferred imaging modality to appreciate the progression of GA, it requires a standard acquisition modality at each exam to ensure comparability, hence a lot of data acquired during routine follow-up cannot be used. On the other hand, the 30${}^{\circ }$ IR imaging is the default mode of fundus image acquisition and hence most patients have such image taken whatever the OCT protocol has been done. Thus, we stick to cSLO with infrared only so that we could have longer and more homogeneous series, and therefore all images are monochromatic and may contain less information than images acquired from other techniques with multiple channels (that are less common for this type of exam and more difficult to find in numbers).

Patients have been followed-up during a few years, hence we have a series of retinal fundus images, sometimes for both eyes (hence the number of series and patients being different in Table 2), showing the progression of the GA. The average number of images in each series is 13. The images are dated from 2007 for the oldest to 2019 for the most recent. All pictures are in grayscale and vary greatly in size, but the most common size is 650 × 650 pixels.

**Figure 2:**
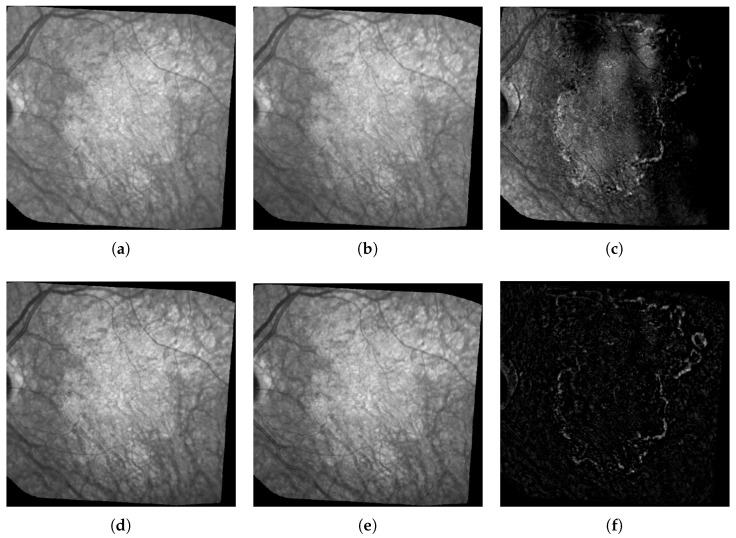
Example of Illumination correction. The three images on the top row represent the two original consecutive images (**a**) and (**b**), and their raw difference in absolute value (**c**); on the bottom row: the same images after illumination correction (**d**) and (**e**), and the new difference (**f**).

As mentioned previously, we notice many imperfections such as blur, artifacts and, above all, non-uniform illumination inside the images and between them (see Figure 2). All images were spatially aligned with i2k software (https://www.dualalign.com/retinal/image-registration-montage-software-overview.php). In every image, the area of useful data does not fill the entire image and is surrounded by black borders. The automatic detection of these black zones in each image gives a mask of the useful data, and the intersection of all masks the common retinal region where changes can be searched for.

We also designed a new method to compensate for illumination distortion between the images (not published yet). This algorithm is based on an illumination/reflectance model and corrects all images of a series with respect to a common reference image. Uneven illumination generally remains present in every processed image (Figure 2), but the smooth illumination distortions are compensated. The calculus of the absolute value of the difference between two consecutive images demonstrates the benefit of this algorithm (Figure 2, last column).

We used five different series of images to evaluate quantitatively our method of change detection: they feature different characteristics in terms of disease progress, lesion shape and size. We developed several user-guided segmentation tools to make the ground truth, based on classical segmentation algorithms: thresholding applied locally on a rectangle defined by the user, parametric active contour model initialized by the user, and simple linear interpolation between points entered by the user. The user can use the most appropriate tool to locally delimit the lesion border, and thus progresses step by step. Local thresholding or active contour algorithm makes it possible to obtain segmentations that depend less on the user than the use of interpolation, which is applied when the two previous methods fail. However, the segmentation remains mostly manual, user-dependent, and tedious. An ophthalmologist realized all segmentation used in our experiments. Finally, the binary change mask between two consecutive images was obtained by subtraction of the segmentation masks.

It is worth noting that the manual segmentation of a single image by expert ophthalmologists takes on average 13 min for a single image, and that many disagreements as to what the ideal segmentation should be arise when comparing the segmentations made by different experts for the same image: In particular, many doctors disagree on which internal changes are interesting or not (and therefore should or should not be in the ground truth), and they may also have different advice for the borders of particularly difficult lesions. For these reasons, and because we kept the results of only a single ophthalmologist, the ground-truth provided for our images have to be taken with caution as they are not always reliable, and, as we will see in later sections, they may feature defects that will affect dices’ indexes computed based on them. Furthermore, for the same reason that it takes a lot of time to produce reliable change maps, our test set is relatively small in size compared to other applications that features much larger data sets, and in particular larger test sets.

## 4. Description of Our Proposed Architecture

Our algorithm is inspired from earlier works from remote sensing [22,26], where the authors applied an unsupervised deep autoencoder to automatically map non-trivial changes between pairs of satellite images to detect meaningful evolutions such as new constructions or changes in landcover while discarding trivial seasonal changes.

In our paper, we use the similarities between satellite images and our medical ARMD eye fundus to adapt their method: both types of images may suffer from lighting issues, noise issues, blurry elements, complex objects present in the images, various time gaps between two images of the same series, and most importantly the common goal of detecting meaningful changes despite all these issues.

While they share similarities, our medical images and satellite images are also quite different: they do not have the same number of channels, they have very different sizes, and the nature of the objects and changes to detect is also quite different. For these reasons, the following subsection will detail how we modified their architecture, and it will explain all steps of our algorithm.

### 4.1. Joint Autoencoder Architecture

As mentioned earlier, in this research, we use a fully convolutional autoencoder. Autoencoders [27] are a type of neural networks whose purpose is to make the output as close as possible to the input. During the learning process, the encoder learns some meaningful representation of the initial data that is transformed back with the decoder. Hence, in a fully convolutional AE, a stack of convolutive layers is applied to the input image in order to extract feature maps (FM) which will then be used to reconstruct the input image.

Usually, AEs with dense layers are used to perform a dimensionality reduction followed by a clustering or segmentation. However, in computer vision, fully convolutional AEs are preferred for their ability to extract textures. Examples of such networks include fully convolutional networks (FCNs) [28] or U-Nets [29]. However, in our case, we do not use Max-pooling layers, and so we keep the same dimensions as the input and only the depth increases.

**Figure 3:**
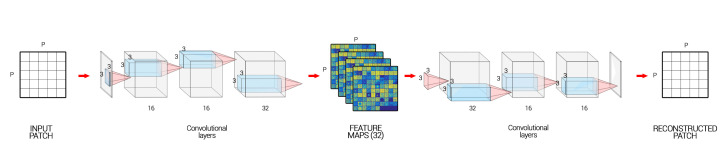
Autoencoder architecture for our algorithm.

Our network (Figure 3) is made of four convolutional layers in the encoder of size 16, 16, 32, and 32, respectively, and in the same way as four convolutional layers of size 32, 32, 16, and 16, respectively, in the decoder side. We apply a batch normalization and a ReLU activation function at each step of the network except for the last layer of the encoder where we only add the L2 normalization, and also for the last layer of the decoder where we apply a Sigmoid function (see in Figure 3).

### 4.2. Algorithm Steps

Our algorithm is made of four steps. We start by dividing the images into several patches. Then, we build the joint autoencoder where it learns how to reconstruct the images, and after we tweak the method by learning the autoencoder to reconstruct not the image itself but the precedent or the future image. The neural networks will learn easily the changes due to the non-uniform illumination or noise but will fail on ARMD progression generating a high reconstruction error (RE), consequently making it possible to detect them. The next subsections will detail some of these steps:

#### 4.2.1. Patches Construction

One of the issues with the retinal fundus images we have is their shape. Indeed, our images are not necessarily square and differ from one set to another. This is why we use an approach based on patches that allows us to manage several sizes of images but also several forms of useful areas thanks to a simple manipulation that we explain right away.

**Figure 4:**
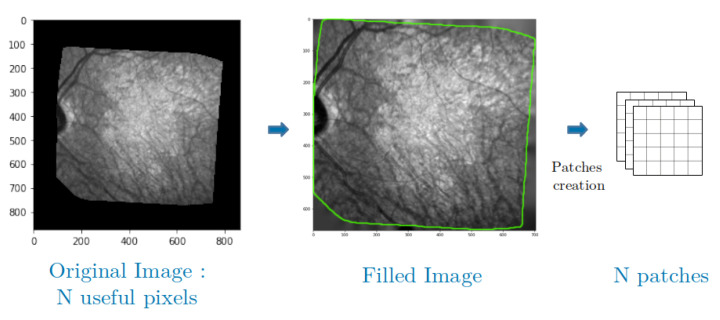
Patches construction, useful pixels are inside the green area.

As mentioned in Section 3, the area of useful data are not rectangular and is generally surrounded by black borders, which can be detected by a simple logic test. As can be seen in Figure 4, we solve this problem by using the Inpainting function of the library *scikit-image* [30] to complete this background. This inpainting function is based on the biharmonic equation [31,32], and it exploits the information in the connected regions to fill the black zones with consistent gray level values. Let us denote by P×P the size of the patches. The image is also padded with $\frac{P}{2}$ pixels along all dimensions and directions before applying the inpainting. Thanks to this operation, we can extract patches from the whole image, i.e., patches centered on every useful pixel, without any cropping, and so we can exploit all our available data without border effects.

#### 4.2.2. Pre-Training

Let us consider a series of *M* images representing the progression of ARMD in a patient’s eye. After the pre-processing and once the images have been aligned and cropped, all images from the same series have the same number of *N* useful patches. From there, to pre-train or network, we sample $\left\lfloor \frac{N}{M}\right\rfloor$ of the patches for every image hence, regardless of the size of the series, we use a total of N patches. This allows us to build a unique autoencoder AE that works for all pairs in the series, and to prevent overfitting.

As an example, for a series of 16 images and 600 × 600 useful patches per image, we would randomly sample $\frac{1}{16}$ of the patches for each image of the series (22,500 patches per image), and use a total of 360,000 patches to pre-train our network.

When processing the patches, our network applies a Gaussian filter in order to weight the pixels by giving more importance to the center of the patch in the RE calculus.

**Figure 5:**
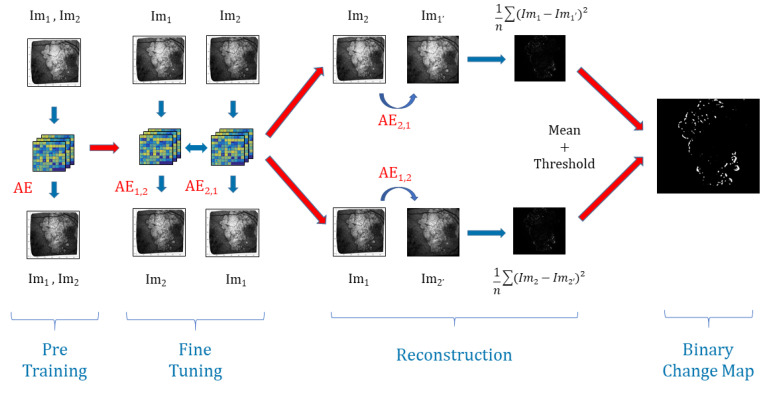
Structure of the algorithm. Example for set of two images $Im_{1}$ and $Im_{2}$ and *n* the number of patches.

During the encoding pass of the AE, the model extracts feature maps of *N* patches of chosen samples with convolutional layers (Figure 5), and then, during the decoding pass, it reconstructs them back to the initial ones.

#### 4.2.3. Fine-Tuning

For every consecutive pair i,i+1 with i∈[[1;M−1]] of images, we are going to build two autoencoders initialized with the weights found in the pre-training part. On one hand, $AE_{i,i+1}$ aims to reconstruct patches of $Im_{i+1}$ from patches of $Im_{i}$ and, on the other hand, $AE_{i+1,i}$ is going to reconstruct patches of $Im_{i}$ from patches of $Im_{i+1}$.

The whole model is trained to minimize the difference between: the decoded output of $AE_{i,i+1}$ and $Im_{i+1}$, the decoded output of $AE_{i+1,i}$ and $Im_{i}$, and the encoded outputs of $AE_{i,i+1}$ and $AE_{i+1,i}$, see Figure 5.

This joint configuration where the learning is done in both temporal directions, using joint backpropagation, has empirically proven to be much more robust than using a regular one-way autoencoder. To optimize the parameters of the model, we use the mean squared error (MSE) of the reconstructed patches.

For the fine-tuning, we stop iterating and running epochs when the MSE of the reconstructed patches stabilizes, as is standard for the training of Deep Learning networks.

#### 4.2.4. Reconstruction and Thresholding

Once the models are trained and stabilized, we perform the image reconstruction. For each pair, we note $Im_{i+1^{\prime }}$ the reconstruction of $Im_{i+1}$ from $Im_{i}$ with $AE_{i,i+1}$ and likewise we note $Im_{i^{\prime }}$ the reconstruction of $Im_{i}$ from $Im_{i+1}$ with $AE_{i+1,i}$. Then, we calculate the reconstruction error RE for every patch between $Im_{i}$ and $Im_{i^{\prime }}$ on one side and between $Im_{i+1}$ and $Im_{i+1^{\prime }}$ on another side. This gives us two images for each pair representing the average REs for $Im_{i^{\prime }}$ and $Im_{i+1^{\prime }}$ that we average to get only one. The model will easily learn the transformation of unchanged areas from one image to the other: changes in luminosity and blurring effects. At the same time, because the changes caused by the disease progression are unique, they will be considered as outliers by the model, and thus will have a high RE. Hence, we apply Otsu’s thresholding [33] that requires no parameters and enables us to produce a binary change map (BCM).

If we sum up, our algorithm uses the strengths of joint auto-encoders to map the light changes, contrast defects, and texture changes from one image to another. This way, most of the image defects and noise issues will be removed. In addition, then, we use the inability of the auto-encoder to predict structural changes in the lesions through time (this algorithm is not designed to do this), as is the inability of the algorithm to generate a high reconstruction error in areas where the lesions have progressed when comparing the encoded reconstructed images with the real images. This way, we have a fully unsupervised way to remove noise and detect changes in the lesions. The full process is explained in Figure 5.

## 5. Experimental Results

### 5.1. Experimental Setting

We chose to compare our methods presented in Section 4.1 with three other methods, all on the preprocessed images. We applied all the methods to three of our series for which we have a ground truth.

The following parameters were chosen for all convolutional layers of our method: kernel size to 3, stride to 1, and padding to 1. The Adam algorithm was used to optimize the models. We set the number of epochs to 8 for the pre-training phase and just 1 for the fine-tuning phase. These parameters were chosen as they are the limit after which the reconstruction error generally does not improve anymore. More epochs during the pre-training phase led to more required epochs to adjust the model to each couple during the fine-tuning phase without much improvements on the results. In addition, more epochs on the fine-tuning phase did not lead to any significant improvement in the results and sometimes resulted in overfitting. Therefore, we fix these parameters both to ensure quality results and to avoid running extra unneeded epochs for both the pre-training and fine-tuning phase.

For both phases, the learning rate was set to 0.0001 and the batch size to 100.

The first method that we use for comparison is a simple subtraction of two consecutive images with an application of Otsu’s thresholding on the result. The second comparison is a combination of principal component analysis (PCA) and K-means algorithm on the difference image proposed by Celik et al. in [21], and we apply it to medical images with blocks of size 5. To finish, we take a Deep-Learning based approach [24] which uses CNN to group similar pixels together with consideration of spatial continuity. This work by Kanezaki et al. was initially made for unsupervised segmentation; consequently, we apply the algorithm to our images and then do the segmentation substractions to get binary change maps. The convolution layers have the same configuration than for our network and we set the parameter for the *minimum number of labels* to 3.

As it is common practice, even for unsupervised algorithms, we assess the results of the different methods using classical binary indexes intended for supervised classification: accuracy, precision, recall, and F1-Score. All formulas are given in Equations (1)–(4), where we used the change areas as the positive class and no change as the negative class. The notations “TP”, “TN”, “FP”, and “FN” are used for true positive, true negative, false positive, and false negative, respectively.

**Accuracy** refers to the proportion of correct predictions made by the model. It is sensitive to class imbalance:

(1) $$Accuracy=\frac{TP+TN}{TP+TN+FP+FN}$$

**Precision** is the proportion of identifications classified as positive by the model that is actually correct:

(2) $$Precision=\frac{TP}{TP+FP}.$$

**Recall** is the proportion of positive results that are correctly identified. It can be thought of as the proportion of progression of the lesion correctly identified:

(3) $$Recall=\frac{TP}{TP+FN}.$$

**F1-Score** also known as the F-Measure, or balanced F Score, is the harmonic mean between the precision and the recall and is computed as follows:

(4) $$F1=\frac{2\times precision\times recall}{precision+recall}$$

### 5.2. Parameters’ Fine-Tuning

The fully convolutional AE model for change detection is presented in Section 4.1.

**Figure 6:**
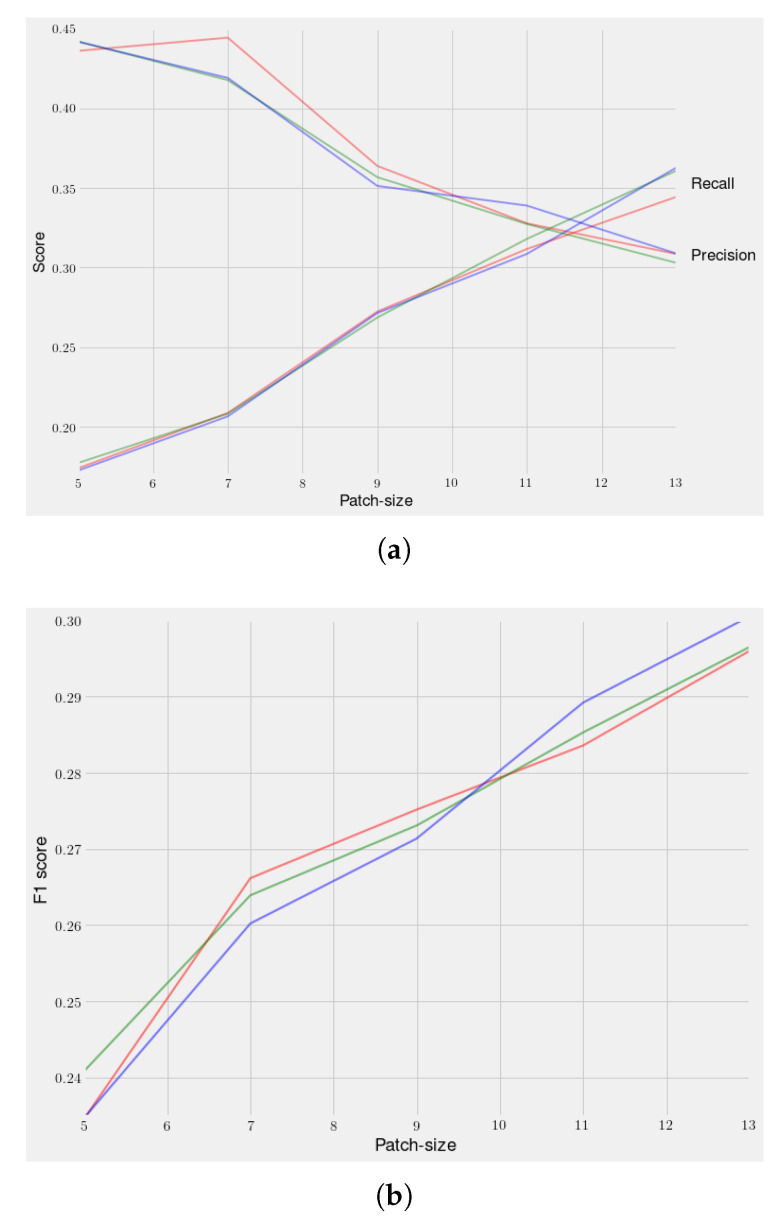
Average recall, Precision, and F1 Score depending on the patch size and sigma: *σ* = 5 *in red* *σ* = 7 *in green* *σ* = 9 *in blue*. (**a**) Recall and Precision depending on the patch size; (**b**) F1 Score depending on the Patch size.

**Figure 7:**
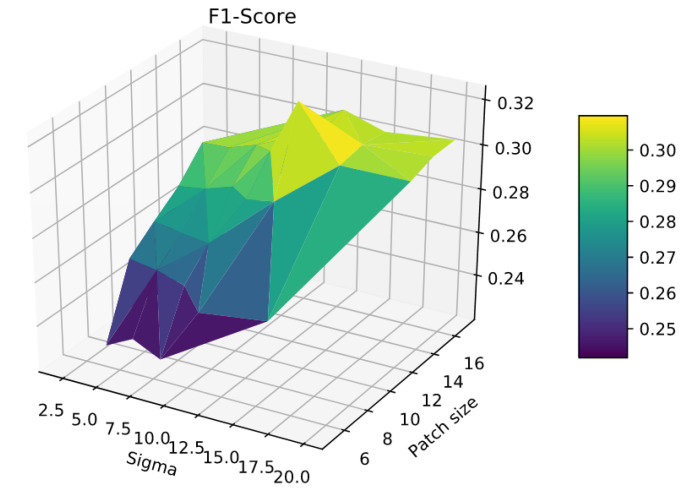
F1-Score as a function of the patch size and the value of sigma *σ* on patient 005.

There are two main parameters in our algorithm. The first one is the the patch size *P*. As we can see in Figure 6, a smaller value of *P* will increase our precision and, on the contrary, a high value of *P* will increase our recall. Thus, the challenge is to find a trade-off value between both scores in order to get the best F1 score possible. In order to improve the performances, we decided to introduce a second parameter: sigma σ. This one refers to Gaussian weights which are applied to the patches to give more importance to central pixels and less to pixels closer to the sides of the patches during the RE loss computation. For each series, we did experiments such as the one shown in Figure 7 to find the best patch size *P* and value σ. In general, we got that a high size of the patch gave us a relatively good recall and the Gaussian weights allow us to regain precision. Two pairs of values came up more often: patch size P=13 and a value σ=12 for patients with larger lesions, and patch size P=7 and a value σ=5 for patients with smaller lesions.

All of the algorithm steps were executed on an Nvidia GPU (RTX TITAN) with 64 GB of RAM and an Intel 9900 k. It took about 20 min for a series of 8 frames with a patch size *P* of 13, with the execution time increasing with it.

### 5.3. Results

**Figure 8:**
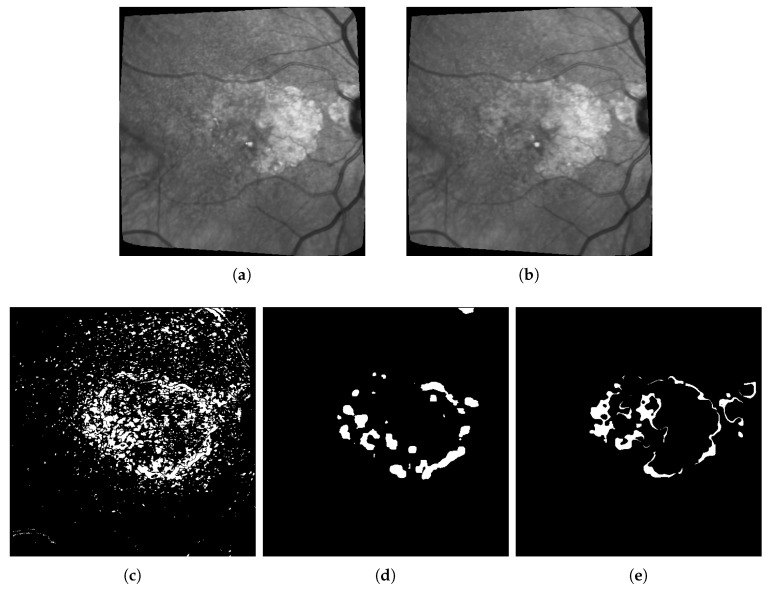
Difference + Otsu thresholding vs. our approach (AE) on patient 003. (**a**) Image at *t* = 0; (**b**) Image at *t* + 3 months; (**c**) Raw difference and Otsu thresholding, F1 score = 0.26; (**d**) Our method, F1 score = 0.36; (**e**) Proposed ground truth.

**Figure 9:**
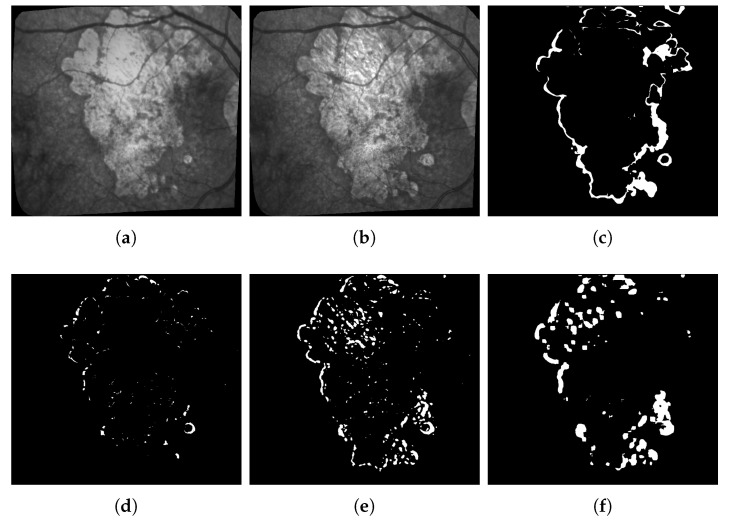
Comparison example of the three methods on patient 005. (**a**) Corrected Image from October 2017; (**b**) Corrected Image from June 2018; (**c**) Proposed ground truth; (**d**) Asako Kanezaki’s approach, F1 score = 0.15; (**e**) Turgay Celik’s approach, F1 score = 0.35; (**f**) Our Fully Convolutional AE, F1 score = 0.4.

**Figure 10:**
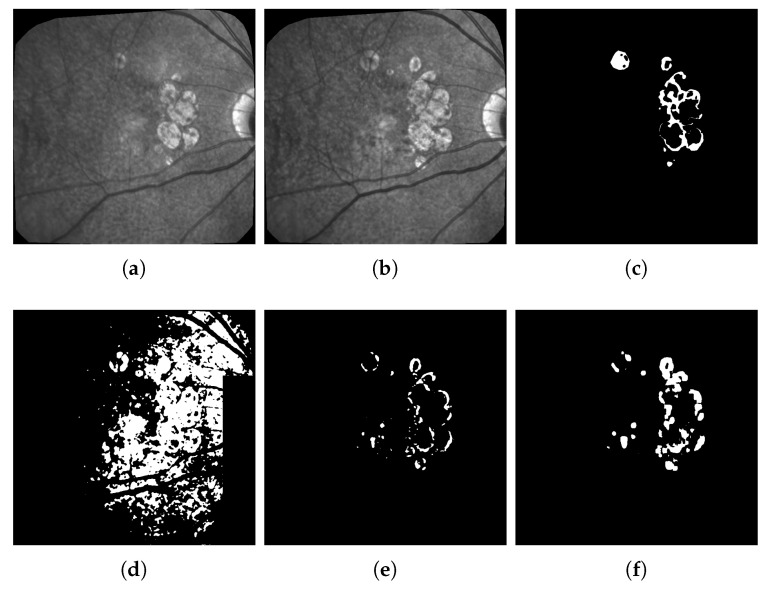
Comparison example of the three methods on patient 001. (**a**) Corrected Image from April 2017; (**b**) Corrected Image from October 2017; (**c**) Proposed ground truth; (**d**) Asano Kanezaki’s approach, F1 score = 0.17; (**e**) Turgay Celik’s approach, F1 score = 0.43; (**f**) Our Fully convolutional AE, F1 score = 0.43.

**Figure 11:**
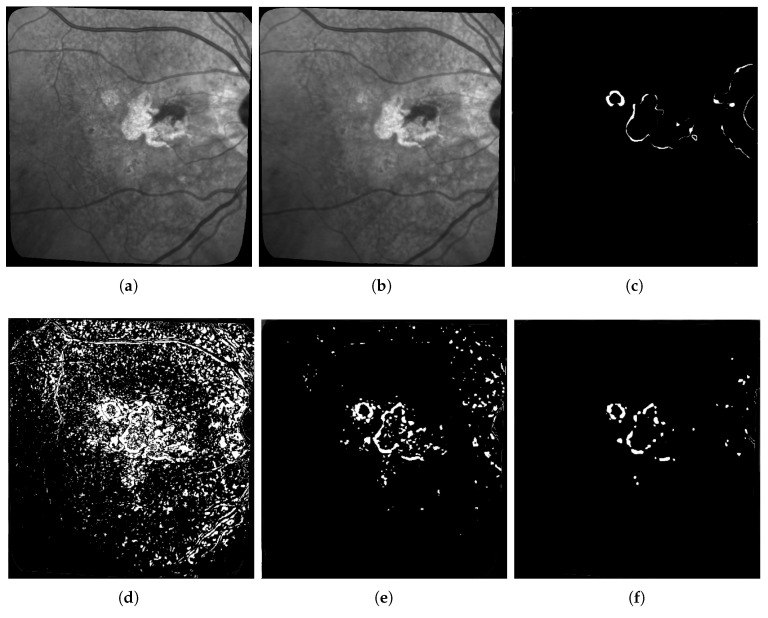
Comparison example of the three methods on patient 010. (**a**) Corrected Image from November 2017; (**b**) Corrected Image from May 2018; (**c**) Proposed ground truth; (**d**) Otsu thresholding, F1 score = 0.05; (**e**) Turgay Celik’s approach, F1 score = 0.253; (**f**) Our Fully convolutional AE, F1 score = 0.38.

The results for patients 1, 3, 5, 10, and 115 are shown in Table 3, as well as Figure 8, Figure 9, Figure 10 and Figure 11 that we added for visual inspection purposes. Additional images for patient 18 are available in some of the figures. Other patients were not added to Table 3 or the figures because we do not have reliable enough change map ground-truths to compute the dice indexes, or because we do not have them at all. Note that the scores presented in Table 3 are for the complete series (15 to 20 pairs per patient), while the scores shown in the figures are for the individual couples of images used in each example. The bottom line of Table 3 shows the weighted mean values for all indexes and all methods for all series.

When looking at Table 3, we can see that the simple difference coupled with Otsu thresholding achieves the best recall results on average that there is no clear winner for the Precision, and that our proposed method on average has the best F1 Score.

Please note that we did not display the Accuracy because of the strong class imbalance, with a large majority of “no change class” pixels leading to results over 85% for Kanezaki’s approach and the simple differentiating with Otsu thresholding, and over 95% for our approach and Celik approach. Since these figures are obviously very biased and given that we are more interested in “change” pixels than “no change” pixels, we did not report them in Table 3 and preferred to focus on commenting on the Precision, Recall, and F1-score that are less affected by class imbalance.

Our interpretation of these results is the following: Otsu thresholding applied to the difference between two images has the best recalls because it detects most real change pixels. However, the binary change map is also very noisy, corresponding to a high number of false positives (wrongly detected changes), which is confirmed by the very low precision score. This can also be observed in Figure 8c and Figure 11d, which are examples of the high number of false positives detected using Otsu thresholding compared with the ground truth in Figure 8c, or our method results in Figure 8e.

In Figure 9, Figure 10 and Figure 11, we compare our approach with the two other algorithms relying on more advanced Machine Learning techniques. First, we can see that, like in Table 3, our approach gets the best F1-score for both patients and pairs of images. Then, we can see that the Kanezaki et al. approach achieves over-segmentation in Figure 9d and under-segmentation in Figure 10d, which highlights that it is more difficult to parametrize properly and may require different parameters for each pair of image, which is not the case for both our approach and the Celik et al. approach. Finally, regarding the comparison between the Celik et al. approach and our proposed method, we can see from Figure 9e,f, Figure 10e,f, and Figure 11e,f that also, like in Table 3, the Celik et al. approach achieves overall good results that are comparable to the ones of our method. However, in the same way that we have better F1-score and accuracy results, the visual results for our methods are also better as the changes we detect in the lesions are cleaner and overall less fragmented into very small elements compared with the ones found by the Celik et al. approach.

When looking at the figures and areas where the changes are detected, we can see that our method finds changes that are more in the peripheral areas of the lesions, while the Celik et al. approach tends to find lots of noisy elements inside existing lesions (see Figure 10e). From a medical point of view and to study the progression of ARMD, we are of course more concerned with lesion growth and therefore with what is going on in the peripheral areas of the lesions, thus giving an advantage to our methods, since it is better at capturing these peripheral changes. However, it does not mean that changes deep inside the lesions have no values and could not be used to better understand the mechanisms of the disease in another study.

### 5.4. Discussion

Overall, we can conclude that both Otsu thresholding and Kanezaki’s approach suffer from risks of over-segmentation detecting a lot of noise, or under-segmentation detecting nothing, both of which are impossible to exploit from a medical point of view. On the other hand, despite somewhat mild recall and precision scores, the Celik approach and our method are visually much better at detecting meaningful changes in ARMD lesions’ structures. Moreover, we can see that, despite the strong class imbalance that we mentioned earlier, our proposed method has a slightly higher F1-Score and finds structures that are visually better and more interesting from a medical point of view since they tend to be more on the outside and at the limits of existing lesions instead of inside them, and are also less fragmented.

To conclude this experimental section, we would like to discuss some of the main weaknesses and limitations of our proposed approach and of this study overall.

**Figure 12:**
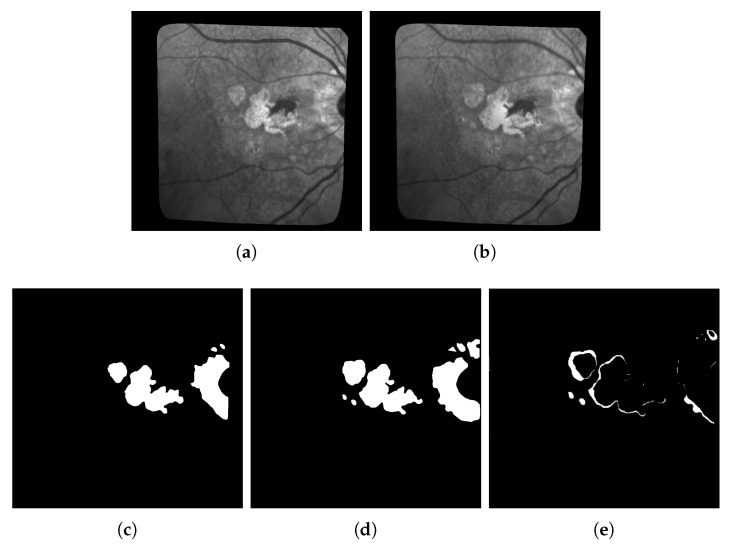
Example of ground-truth build for patient 010 based on two consecutive masks of segmented lesions at time *t* and t+1: All changes inside the lesions, textural or otherwise, are ignored. (**a**) Image *t*; (**b**) Image *t* + 1; (**c**) segmentation mask *t*; (**d**) segmentation mask *t* + 1; (**e**) Ground truth built from Mask *t* and t+1.

The first limitation that is not inherent to our approach is the difficulty to get accurate ground-truths to assess the quality of the results (hence why unsupervised learning should be preferred). In particular, all the ground-truths we have completely ignore possible textural changes within existing areas of geographic atrophy, and they don’t always have the level of accuracy we hope for when it comes to subtle small changes in the lesions. This is due to the fact that most of these ground truths are built by subtracting masks of segmented lesions during two consecutive exams as shown in Figure 12: Doing so results in ground-truths that ignore most of the changes happening inside the lesions. It is worth mentioning that series with larger lesions are more affected by this issue as these lesions are more likely to have internal changes.

**Figure 13:**
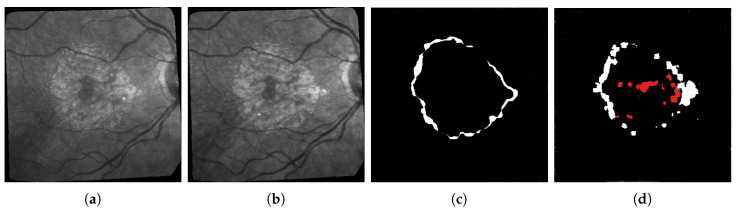
Example of a segmentation in (d) where all changes detected in red will be considered false positive since the ground truth does not consider changes within existing lesions regardless of if they are structural of textural. (**a**) Image of patient 018 at time *t*; (**b**) Image of patient 018 at time *t* + 1; (**c**) Proposed Ground truth; (**d**) Proposed segmentation.

This explains some of the low dice scores from Table 3 since in many cases there are internal changes (textural, structural, or both) happening within ARMD lesions, and some of them will be detected by the algorithms used in this paper. However, almost all of these changes detected inside the lesions will be classified as false positive since they are not present on the ground truth. One example of such issue is shown in Figure 13 where all pixels in red in sub-figure (d) will be classified as false positive despite some of them being actual changes (seeing some internal structural changes is possible by zooming in on sub-figures a and b).

The second obvious weakness of our approach is also related to the difficulty of finding reliably annotated data and ground-truth. Because it is difficult to find them, we propose an unsupervised approach. In addition, unfortunately, unsupervised approaches are known to produce results that are less impressive than their supervised counterparts. A fully unsupervised framework which has the advantage that it does not require any annotated data to be trained—which is a real strength when very few are available—but has the weakness that its performances are weaker. In future works, we hope to refine our method so that we can improve the quality indexes a bit, but, even with better ground-truths, we expect the performances to remain limited so long as the framework is fully unsupervised.

A third limitation that can be mentioned and comes more from the data and experimental protocol than the methods is the following: Given that our method is fully unsupervised, and since we use change maps made by experts ophthalmologists that are not always reliable due to disagreements between experts on what is an interesting change or not, we have both a test set problem (as mentioned before), and a choice of metric problem as dice indexes while commonly used are probably not ideal to evaluate unsupervised methods, especially when there is uncertainty on the quality of the expert change maps. Two solutions that we plan to use on our future works are the following:

- To have several experts rating the proposed change maps, and use the average mark as a quality index. This method has the advantage that it is the fairest, but it is inconvenient that it is very time consuming and does not scale with large datasets as it takes on average 13 min for an ophthalmologist to do a quality segmentation on a single image.
- Using unsupervised quality indexes alongside the accuracy and the three dice indexes that we already use, so that we have a more fair evaluation of all methods. While this has the advantage of scaling well, it is probably a weaker quality argument than supervised indexes and experts’ ratings for an application in the field of medicine.

The fourth limitation of our method that can be pointed out is that, as it stands, our algorithm does not yet achieve the goal of predicting how the pathology evolves: Our method detects changes and how the ARMD lesions evolved from one image to the next, but it does not provide any growth model or any interpretation of why it grew this way. Furthermore, while our algorithm detects changes fairly well, it is yet unable to predict future changes and therefore to tell in advance how the lesions might evolve on the short or long term for a given patient. While prediction was not the goal of the method proposed in this paper, it is certainly a future evolution that we are interested in. In fact, we hope that we will be able to use the results provided by our method on how the lesions grow and change from one image to the next to build predictive models based on this information. Some leads on approaches with which to combine our algorithm include other deep learning approaches such as long short term memory [34], gated recurrent units [35], or generative adversarial networks [36], all of which have shown to be useful for time series prediction or long term predictions.

Finally, even if our work does not lead to a huge leap forward in result quality due to various issues that we previously mentioned, the fact that we proposed a deep learning architecture will make it a lot easier in the future to couple our approach with predicting architectures such as the ones we just mentioned.

## 6. Conclusions

In this paper, we have presented a new fully unsupervised deep learning architecture based on a joint autoencoder that detects the evolution of ARMD lesions in an eye fundus series of images. With a pre-cleaning of the series to remove as many lighting issues as possible, our proposed method is based on an auto-encoder architecture that can detect non-trivial changes between pairs of images, such as the evolution of a lesion, while discarding more trivial changes such as lighting problems or slight texture changes due to different image angles. Our proposed method was applied to three real sets of images, and was compared with three methods from the state of the art. Despite mild F1-Score results due to various issues, our method has been shown to give good enough results for a fully unsupervised algorithm and to perform better than the other methods from the state of the art, and may prove useful to assist doctors in properly detecting the evolution of ARMD lesions by proposing a first raw segmentation of the evolution.

While our results are not perfect and cannot yet be used for a fully automated diagnosis, it is obvious to us that our proposed algorithm may prove useful to assist doctors in properly detecting the evolution of ARMD lesions by proposing a first raw segmentation of the evolution that is a lot better than what can be done with the existing methods.

In our future works, our priority will be to solve our ground-truth and test set issues by trying to have more experts producing and rating our results. This is an important pre-requisite as we plan on working on approaches that can work on full time series rather than pairs of images. This would also require both better lighting correction algorithms but may lead to more interesting models to predict the evolution of ARMD. Developing long-term prediction algorithms is another goal of ours that could be achieved using a combination of this work on longer series with other deep approaches that are more adapted for prediction.

Finally, as there are other modalities of images available to study ARMD, some of which have colors, but with different resolutions, one of our other goals would be to combine several of these types of images to globally improve our prediction scores. This future work of combining images with different scales, alignments, and color bands shall prove to be very challenging and will hopefully yield more interesting results while still using a fully unsupervised approach.

## Figures and Tables

**Table 1:** Summary of the state-of-the-art methods for change detection that are mentioned in this work.

Authors	Supervised	Images Used	Algorithm	Application
Troglio, Napo et al. [11,12]	No	Pairs	K&I Thresholding	ARMD
Marrugo et al. [6]	No	Pairs	Image correction	ARMD
Köse et al. [5]	semi	Individual	Raw segmentation	ARMD
Ramsey et al. [4]	No	Individual	Fuzzy C-Means	ARMD
Hussain et al. [13]	Yes	Individual	U-Nets	ARMD
Burlina et al. [14,15]	Yes	Individual	pre-trained CNN	ARMD
Kanezaki et al. [24]	No	Individual	CNN	Image Processing
Sublime et al. [26]	No	Pairs	Joint-AE & KMeans	Remote Sensing
Celik et al. [21]	No	Pairs	PCA & KMeans	Remote Sensing
Our Method	No	Pairs	Joint-AE	ARMD

**Table 2:** Description of the data.

Number of patients	15
Number of image time series	18
Average number of images per series	13
Total number of images	336
Acquisition period	2007–2019
Average time between two images	6 months

**Table 3:** Results and comparison of the different approaches. It contains the means of the recall, the precision, and the F1 score for each time series. For each patient, the best dice results are in bold.

Patient ID	Method	Authors	Recall	Precision	F1 Score
001 *15 images*	Diff + Otsu	-	**0.68**	0.11	0.16
	CNN	Kanezaki et al.	0.32	**0.29**	0.18
	PCA + KMeans	Celik et al.	0.48	0.28	**0.3**
	Joint-AE	Our method	0.44	0.21	0.26
003 *5 images*	Diff + Otsu	-	**0.55**	0.1	0.17
	CNN	Kanezaki et al.	0.2	0.27	0.07
	PCA + KMeans	Celik et al.	0.24	**0.33**	0.27
	Joint-AE	Our method	0.29	0.28	**0.28**
005 *8 images*	Diff + Otsu	-	**0.46**	0.2	0.26
	CNN	Kanezaki et al.	0.2	**0.43**	0.21
	PCA + KMeans	Celik et al.	0.26	0.37	0.28
	Joint-FCAE	Our method	0.33	0.34	**0.32**
010 *6 images*	Diff + Otsu	-	**0.68**	0.06	0.10
	CNN	Kanezaki et al.	0.32	0.03	0.05
	PCA + KMeans	Celik et al.	0.47	0.23	0.29
	Joint-FCAE	Our method	0.38	**0.35**	**0.36**
115 *9 images*	Diff + Otsu	-	**0.53**	0.25	0.29
	CNN	Kanezaki et al.	0.33	0.15	0.16
	PCA + KMeans	Celik et al.	0.24	0.38	0.25
	Joint-FCAE	Our method	0.33	**0.39**	**0.34**
Total *(patients’ mean—43 images)*	Diff + Otsu	-	**0.58**	0.16	0.20
	CNN	Kanezaki et al.	0.27	0.27	0.14
	PCA + KMeans	Celik et al.	0.33	**0.32**	0.27
	Joint-AE	Our method	0.35	**0.32**	**0.31**

## Abbreviations

The following abbreviations are used in this manuscript:

AE	Autoencoder
ARMD or AMD	Age Related Macular Degeneration
BCM	Binary Change Map
CNN	Convolutional Neural Networks
cSLO	confocal Scanning Laser Ophthalmoscopy
FAF	Fundus Autofluorescence
GA	Geographic Atrophy
IR	Infrared
OCT	Optical Coherence Tomography
PCA	Principal Component Analysis
RE	Reconstruction Error
RPE	Retinal Pigment Epithelium
